# Multicolor Visual Detection of Deoxynivalenol in Grain Based on Magnetic Immunoassay and Enzymatic Etching of Plasmonic Gold Nanobipyramids

**DOI:** 10.3390/toxins15060351

**Published:** 2023-05-23

**Authors:** Rui Guo, Yue Ji, Jinnan Chen, Jin Ye, Baoxia Ni, Li Li, Yongtan Yang

**Affiliations:** 1 Academy of National Food and Strategic Reserves Administration, No.11 Baiwanzhuang Str., Xicheng District, Beijing 100037, China; gr@ags.ac.cn (R.G.); jiiyue1998@163.com (Y.J.); cjn@ags.ac.cn (J.C.); yj@ags.ac.cn (J.Y.); nbx@ags.ac.cn (B.N.); ll@ags.ac.cn (L.L.); 2 College of Food Science and Engineering, Jilin Agricultural University, Changchun 130118, China

**Keywords:** deoxynivalenol, multicolor visual detection, magnetic immunoassay, gold nanobipyramids, enzymatic etching

## Abstract

In this study, a multicolor visual method based on a magnetic immunoassay and enzyme-induced gold nanobipyramids (Au NBPs) etching was developed for deoxynivalenol (DON) detection. The magnetic beads modified with high affinity DON monoclonal antibodies were used as a carrier for target enrichment and signal transformation and the Au NBPs with excellent plasmonic optical properties were served as enzymatic etching substrates. The oxidation state TMB, which was generated through catalysis of horseradish peroxidase (HRP), induced the etching of plasmonic Au NBPs, resulting in the longitudinal peak blue-shift of local surface plasmon resonance (LSPR). Correspondingly, Au NBPs with various aspect ratios displayed a variety of individual colors which were visualized by the naked eye. The LSPR peak shift was linearly related to the DON concentration in the range of 0~2000 ng/mL and the detection limit was 57.93 ng/mL. The recovery for naturally contaminated wheat and maize at different concentrations ranged from 93.7% to 105.7% with a good relative standard deviation below 11.8%. Through observing the color change in Au NBPs, samples with overproof DON could be screened preliminarily by the naked eye. The proposed method has the potential to be applied in on-site rapid screening of mycotoxins in grain. In addition, the current multicolor visual method only used for the simultaneous detection of multiple mycotoxins is in urgent need of a breakthrough to overcome the limitation of single mycotoxin detection.

## 1. Introduction

Deoxynivalenol (DON), a trichothecene mycotoxin produced mainly by Fusarium graminearum and Fusarium culmorum, is commonly found in contaminated grains such as wheat, barley, and maize [1,2]. DON contamination is widespread worldwide, throughout grain growing, storing, transporting, and processing. Due to its inherent stability, DON is difficult to remove during circulation. If ingested, DON contaminated food will cause people to suffer from anorexia, vomiting, fever, diarrhea, immunotoxicity, and cardiotoxicity, which will lead to hematopoietic dysfunction and death in severe cases [3,4]. Therefore, early warning and dynamic monitoring of DON during grain growth and storage are effective ways to prevent food being contamination. Thus, developing a highly sensitive, highly specific, and portable method for DON analysis is a challenge in the field of food safety today, which attracts considerable interest from researchers.

Traditional DON detection methods mainly depend on chromatographs coupled with diverse detectors, including high-performance liquid chromatography [5,6], gas chromatography–mass spectrometry [7], and liquid chromatography–tandem mass spectrometry [8,9]. Chromatographic techniques display high sensitivity and accuracy with increased analytical efficiency but are limited in the laboratory by expensive instruments, tedious sample pretreatment, and the requirement of professional operators. Whereafter, alternative approaches have emerged with the advantages of convenient operation and fast response time, such as enzyme-linked immunosorbent assay [10], gold immunochromatography assay [11], time-resolved fluoroimmunoassay [12], surface plasmon resonance [13,14], surface-enhanced Raman spectroscopy [15], and colorimetric assay [16,17]. Particularly, a colorimetric assay is suited for on-site detection because the result can be observed by the naked eye through various color changes. The universal colorimetric method only presents monochromic intensity change, which is not quite satisfactory to be observed by the naked eye. Therefore, it is necessary to establish a facile and reliable colorimetric method to fulfill on-site DON rapid screening in grain.

In recent decades, plasmonic nanomaterials have been widely applied in biotechnology, electronics, optical devices, sensors, and catalysis due to their unique physical and chemical properties [18,19]. Among them, noble-metal nanomaterials have been extensively applied to plasmonic biosensing due to the local surface plasmon resonance (LSPR) absorption of light resulting from the oscillation of the free electrons [20,21,22]. Au nanomaterials with various sizes, morphologies, and compositions present excellent prospects in colorimetric assays since the LSPR peak shift. In comparison with a conventional monochromic assay, the plasmonic colorimetric method due to the LSPR peak shift displays abundant colors and improved resolution upon visual inspection [23]. Distinctively from other nanostructures, Au nanobipyramids (Au NBPs) with two sharp apexes possess significantly enhanced localized electromagnetic fields and narrower plasma linear widths as well as higher refraction indexes; the aspect ratio of Au NBPs is more easily changed during growing or etching. Due to the improvement in the Au NBPs synthesis strategy in terms of the high homogeneity and yield, the applications in highly sensitive visual detection have been gradually explored [24,25,26,27,28].

In this work, we report a plasmonic colorimetric method for DON visual detection based on magnetic immunoassays and enzymatic etching of Au NBPs. The immunomagnetic beads (IMBs) were used to recognize and enrich target mycotoxin from samples by specific affinity. Through magnetic separation, the matrix interference was reduced and the selectivity of DON detection was significantly improved. HRP-linked DON antigen was competitively connected to IMBs and catalyzed TMB to the oxidation state TMB^2+^. Then, the produced TMB^2+^ was served as an etchant for plasmonic Au NBPs and induced a vivid color response. The relationship between DON content and the LSPR shift of Au NBPs was established and the result could be readout straightforwardly by the naked eye. The detection process integrates an automated multiplex reaction system and the adsorption, separation, transfer, washing, and catalytic reaction were carried out in sequence and 24 samples could be tested simultaneously in 40 min. Compared with the current DON detection methods, the proposed method is automatic, labor-saving, and efficient, making it a portable platform for large-scale mycotoxin screening.

## 2. Results and Discussion

### 2.1. Principle of Multicolor Visual Strategy for DON Detection

**Figure 1:**
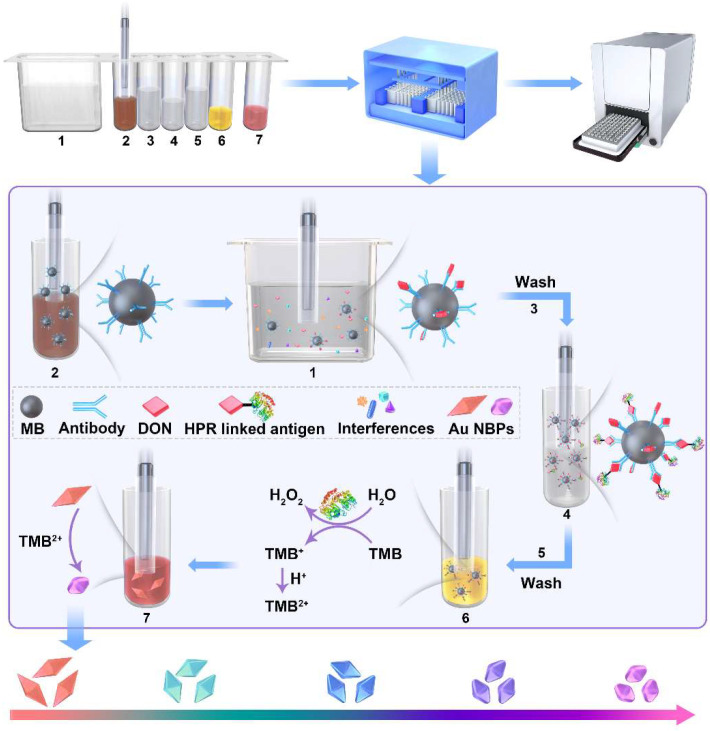
Schematic diagram of multicolor visual detection of DON.

As shown in Figure 1, the detection system uses IMBs as carriers for target enrichment and transfer, HRP-linked DON antigen as a catalyst, and Au NBPs as an etching substrate for multicolors. DON mAb was immobilized on NHS-activated MBs through the covalent linkage between amino and carboxyl. Due to the specific affinity of antigen to the IMBs, target DON and HRP-linked DON antigens were connected to IMBs successively. The HRP-linked IMBs were capable of enzymatic activity to catalyze the oxidation reaction between TMB and H_2_O_2_ and generate TMB^2+^, which can be used as an oxidant to etch Au NBPs. During this process, the sharp tips of the unique pentagonal pyramids at the apexes were shortened, accompanied by the blue-shift of the LSPR peak and the color transition of Au NBPs colloid solution. If the sample was contaminated by DON, the partial active site of IMBs would be occupied. DON content was negatively correlated with the catalytic activity of IMBs and the quantitative relationship was established. The detection result can be observed by the naked eye and accurately quantitated by the LSPR band measured via the microplate spectrophotometry.

### 2.2. Characterization of Au NBPs

**Figure 2:**
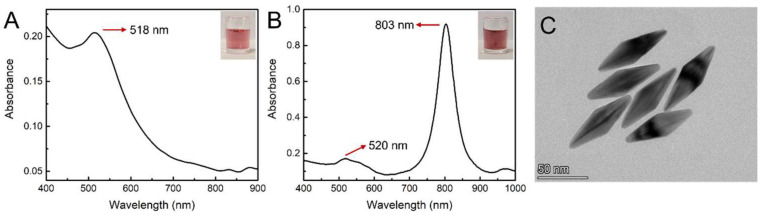
UV–vis spectrum and corresponding optical photographs of Au seeds (**A**) and Au NBPs (**B**). (**C**) TEM images of Au NBPs.

Au NBPs were synthesized based on a seed-mediated single-growth step method, which avoided tedious procedures and exhibited superior synthetic yields. The Au seeds and Au NBPs were characterized by UV–vis spectroscopy and corresponding optical photographs were taken by a smartphone camera. As shown in Figure 2A, the UV–vis absorption peak of Au seeds is located at 518 nm and the colloidal solution displayed a red color. As shown in Figure 2B, the resulting Au NBPs display a strong and narrow longitudinal plasmon resonance at 803 nm and a transversal plasmon band at 520 nm, respectively. The intensity of the longitudinal peak is 5.4-fold over that of the transversal peak, indicating a high yield of homogeneous Au NBPs. The microscopic morphology of Au NBPs was imaged by TEM, as shown in Figure 2C. The Au NBPs showed a symmetrical pentahedron structure with an average length of 77 ± 3 nm in the longitudinal direction and 24 ± 1 nm in the transverse direction. The uniform size and good monodispersity were satisfactory. The Au NBPs with various aspect ratios were synthesized by adjusting the additional amount of Au seed solution and the UV–vis spectrum was displayed in Figure S1A. The LSPR peak of Au NBPs is blue shifted gradually with the increasement of Au seeds, as shown in Figure S1B. The Au NBPs with desired LSPR peaks can be synthesized according to the linear equation.

### 2.3. Feasibility of the DON Detection

**Figure 3:**
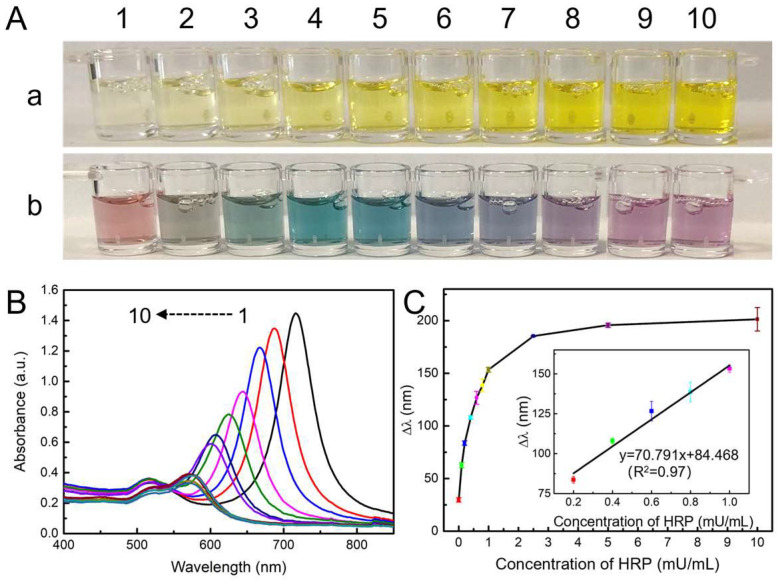
(**A**) Optical photographs of produced TMB^2+^ solutions before (**a**) and after (**b**) the addition of Au NBPs. (**B**) The UV–vis absorption spectra of Au NBPs are etched by various concentrations of TMB^2+^. From 1–10, concentrations of HRP were 0, 0.1, 0.2, 0.4, 0.6, 0.8, 1.0, 2.5, 5.0, and 10.0 mU/mL, respectively. (**C**) Plot of Δλ vs. concentration of HRP. Inset: the linear relationship between Δλ and the concentration of HRP from 0.2 to 1.0 mU/mL.

To testify to the feasibility of the multicolor detection system, the oxidation ability of TMB^2+^ to quantitative etching Au NBPs was first evaluated. As the concentration of HRP increased from 0 to 10 mU/mL, the produced TMB^2+^ displayed a yellow color with various intensities (Figure 3A(a)). The absorbance at 452 nm was positively correlated with the HRP concentration in the range of 0 to 1.0 mU/mL (Figure S2). However, the monochromic intensity was difficult to be discriminated by the naked eye. It has been demonstrated that the etching of Au NBPs will change the aspect ratio, which resulted in a distinctive multicolor tonality evolution. Meanwhile, owing to the sharp apexes, Au NBPs possess higher local electric-field enhancements and superior LSPR properties. The aspect ratio is easier to be changed and the color response is predictable to be improved. Hence, Au NBPs were selected as chromogenic response substrates. As shown in Figure 3A(b), after adding Au NBPs, the yellow solution showed abundant colors changed from red to gray, celadon, cyan, blue, navy, violet, purple, and mauve. The UV–vis absorption spectra displayed that the longitudinal LSPR peak of Au NBPs blue shifted from 716 nm to 572 nm and the corresponding absorbance intensity decreased gradually. While the transverse LSPR peak was almost constant at 520 nm with decreased intensity (Figure 3B). The phenomenon could be ascribed that the etching reaction preferentially occurred on the sharp apexes of Au NBPs. The LSPR peak shift (Δλ) was linearly related to the HRP concentration in the range of 0 to 1.0 mU/mL (Figure 3C). The results proved that the principle of enzymatic etching Au NBPs to produce multi colors was feasible.

**Figure 4:**
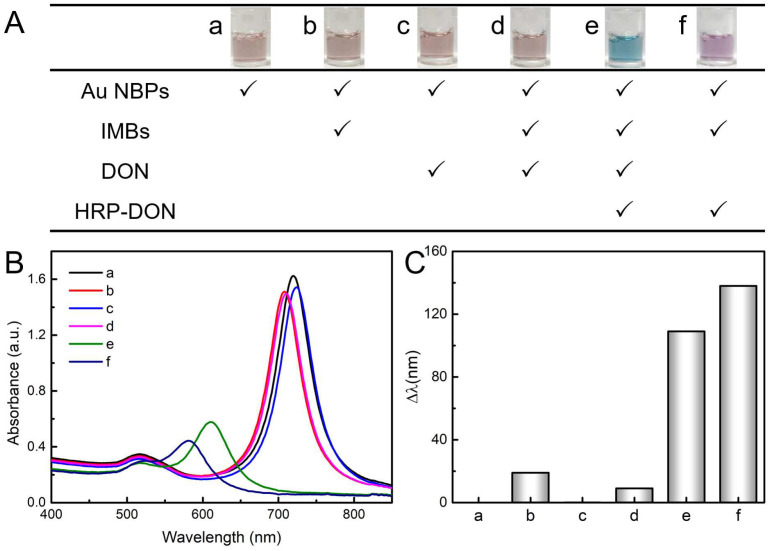
Feasibility of the DON detection principle. (**A**) Table of reagents added to each tube. Inset: optical photographs of the corresponding color tonalities of Au NBPs colloidal dispersion. UV–vis absorption spectra (**B**) and Δλ (**C**) of Au NBPs under the corresponding conditions.

Thereafter, the visual DON detection strategy based on the immunoaffinity and enzymatic etching Au NBPs was explored following the program of reagent addition, as shown in Figure 4A, and the insets exhibit the color change in Au NBPs. It is observed that the Au NBPs displayed a constant color with the addition of MBs and DON. However, the color changed dramatically with the addition of HRP-linked DON antigen. Interestingly, the solution showed blue color in the presence of DON (Figure 4A(e)) and a mauve color in the absence of a DON (Figure 4A(f)), the results can be easily distinguished by the naked eye. The mimic peroxidase function of magnetic nanomaterials might cause a false positive result. Nevertheless, the negligible peak shift had no effect on the detection signal (Figure 4A(b,d)) with the 0.15 mg/mL IMBs. Correspondingly, the UV–vis absorption spectra and longitudinal LSPR peak shift (Δλ) were displayed in Figure 4B,C. The LSPR peak barely moved in a to d and the Δλ in e and f are 109 nm and 138 nm, respectively. The HRP-linked DON antigen was the key to inducing the catalytic reaction between TMB and H_2_O_2_ and producing oxidation reagent TMB^2+^ for quantitative etching of Au NBPs. Once the IMBs capture DON in the sample, the amount of HRP-linked DON antigen binding onto IMBs will be reduced, resulting in a decrease in Δλ. These results indicate that the visual DON detection strategy based on the immunoaffinity and enzymatic etching Au NBPs is viable.

### 2.4. Optimization of Experimental Conditions

Experimental conditions were optimized to achieve the best analytical performance, including the reaction time of catalytic and etching procedures and the concentrations of CTAB. In this work, TMB^2+^ was used as an etching agent to oxide Au NBPs to generate Au(I) and its yield was affected by the catalytic reaction between TMB and H_2_O_2_. As shown in Figure S3A, the absorption intensity of the catalyst increased gradually and then reached a parallel level at 8 min. Thus, the catalytic time was set at 5 min. Figure S3B showed the effect of TMB^2+^ etching reaction time on Δλ, where it enhanced rapidly in the initial 2 min and keep growing in the following 14 min. Considering the color response of Au NBPs, 5 min of the etching time was selected. Moreover, CTAB plays a vital role in Au NBPs etching because it can lower the redox potential of Au(I)/Au by formatting an ion association compound. As CTAB increased from 0 to 0.06 M, the absorption peak of TMB^2+^ in 452 nm decreased and Δλ of Au NBPs increased sharply (Figure S3C,D). Nevertheless, Δλ showed a slight decrease as CTAB increased from 0.06 to 0.14 M. It is ascribed that the higher viscosity of CTAB impeded the uniform mixing of TMB^2+^ and the Au NBPs. Hence, the optimal CTAB concentration was selected as 0.06 M.

### 2.5. Performance Evaluation of the Proposed Method

**Figure 5:**
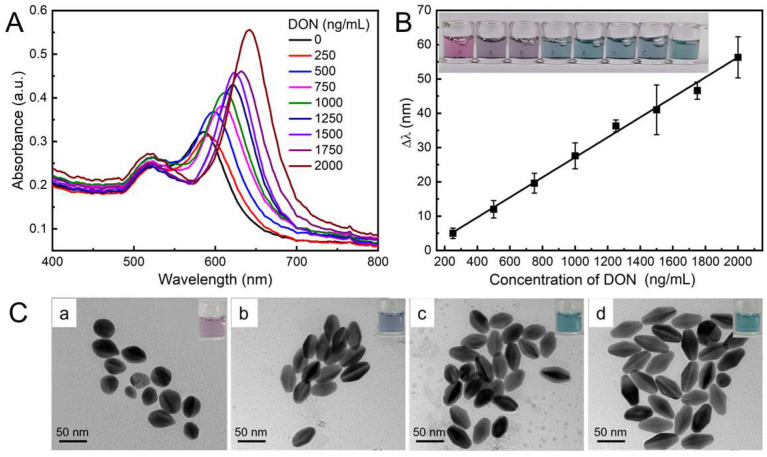
(**A**) UV–vis absorption spectra of Au NBPs in response to various concentrations of DON from 0 to 2000 ng/mL. (**B**) Linear relationship between Δλ and the concentration DON, the insets show the color tonality variations. (**C**) Typical TEM images of Au NBPs in the presence of DON from 0 (**a**), 500 (**b**), 1000 (**c**), and 2000 ng/mL (**d**), respectively. The insets show the optical photographs of the corresponding colloidal solutions.

Under the optimal experimental conditions, DON with various concentrations were introduced to evaluate the analytical performance. As illustrated in Figure 5A, the longitudinal LSPR peak of Au NBPs exhibited red shift with the increase in DON. As Figure 5B shows, there is a linear relationship between Δλ and the concentration of DON ranging from 0 to 2000 ng/mL. The calibration equation is Δ*λ* = 0.029 [*C*_DON_(ng/mL)] − 2.012 (*R*^2^ = 0.993) and the limit of detection (LOD) was calculated to be 57.93 ng/mL based on the rule of 3*σ*/*S* (*σ* is the standard deviation of control sample, *S* is the slope of the standard curve). Correspondingly, the color of Au NBPs changed from mauve to purple and cyan as shown in the insets. According to the discerned color tonality evolution, DON exceeding 1000 ng/mL can be easily distinguished at a glance by the naked eye. The proposed multicolor visual immunoassay exhibited a wide linear range and sensitive analysis, as well as a time-saving automatic operation which possessed a particular impact on the visual semi-qualitative detection of DON.

The design principle of this experiment is to establish the relationship between the concentration of DON and the color change in Au NBPs. Generally, the color of Au NBPs colloidal solution is related to the morphology and size of Au NBPs. In order to further explore the morphology and color change in Au NBPs during DON detection, typical TEM images were used to characterize the etching processes with representative concentrations of DON including 0, 500, 1000, and 2000 ng/mL. As manifested in Figure 5C(a), Au NBPs showed a quasi-spherical structure in the absence of DON. As the concentration of DON increased, the transverse length of Au NBPs remained constant while the longitudinal length gradually increased, which is attributed to the etching reaction preferentially occurring at the tips of Au NBPs (Figure 5C(b–d)). The inset optical photographs further verified the color change in the corresponding solution. The results revealed that the color change and LSPR peak shift of the colloidal solution were caused by the morphology variation of Au NBPs, which was induced by introducing various amounts of DON.

The specificity is a significant parameter for evaluating the performance of the proposed method. To assess the specificity of the multicolor visual detection, the effects of potential interferences including AFB_1_, AFM_1_, ZEN, and OTA were investigated. The optical photograph in Figure S4A showed that there was no apparent difference between these interferences and the blank control, and only the addition of DON caused a significant color change. The values of Δλ were calculated as shown in Figure S4B; only DON contributed to the remarkable shift in the LSPR peak. The results indicated that the designed assay can selectively detect DON in the presence of other mycotoxins. The performance of the developed sensor is compared with the ones already reported in the literature, as shown in Table S2 in the Supplementary Materials.

### 2.6. Analytical Application for Actual Samples

**Figure 6:**
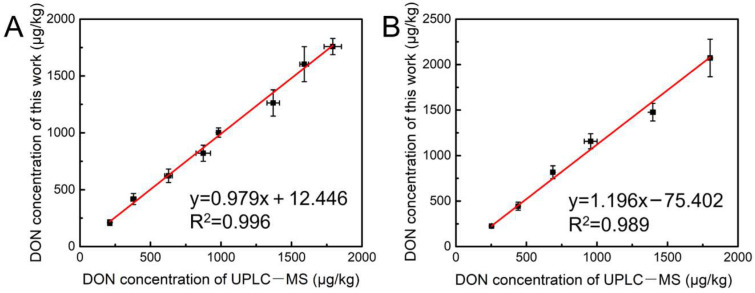
Comparison of determination results of naturally contaminated samples of wheat (**A**) and maize (**B**).

The reliability of the developed method was demonstrated by detecting naturally contaminated samples of wheat and maize, and the results were listed in Table 1. It showed that the recovery of wheat was 95.5–105.7% with RSD of 4.06–11.80% and the recovery of maize was 93.7–107.5% with RSD of 5.15–10.03%, proving that the method provided good accuracy and precision. The results were verified by ultra-performance liquid chromatography–mass spectrometry (UPLC–MS). As shown in Figure 6, the regression analysis between the two methods indicates that the results from the proposed method agreed well with those obtained from the UPLC–MS.

## 3. Conclusions

In summary, we develop a multicolor sensing method based on the excellent optical properties of Au NBPs to realize the visual detection of DON. To our knowledge, enzyme-induced etching of plasmonic Au NBPs and magnetic immunoassay have been integrated for the first time to detect DON through multicolor variation. Compared with the traditional DON detection method, this proposed strategy is intuitive, convenient, and efficient. Through the immunoaffinity reaction, the target DON and HRP-linked DON antigen can be captured sequentially and IMBs with enzymatic activity were acquired to generate TMB^2+^ as the etchant. Via the utilization of Au NBPs as signal reporters, a distinctive and vivid colorimetric readout for the naked eye was provided for rapid screening samples containing overproof DON. The reaction process was conducted automatically with a magnetic-controlled program and 24 samples could be tested simultaneously in 40 min, thus the tedious manual manipulation was avoided. The applicability of the method was also approved by analyzing the naturally contaminated grain samples of wheat and maize with satisfactory results. It makes the multicolor visual detection greatly valuable in mycotoxins on-site rapid screening during grain purchase and storage. Moreover, the Au NBPs is possible to self-assembling into photonic crystal for signal enhancement through coupling emission.

## 4. Materials and Methods

### 4.1. Materials and Chemicals

N-Hydroxysuccinimide-functionalized magnetic beads (NHS-MBs) were bought from Beaver Bioscience Inc. (Suzhou, China). DON monoclonal antibodies (mAb) were purchased from Chuangpu Biotechnology Co., Ltd. (Wuxi, China). HRP-linked DON antigen was obtained from Jiesheng Jiekang Biotechnology Co., Ltd. (Wuxi, China). TMB two-component substrate solution and HRP were bought from Beijing Solarbio Life Science Co., Ltd. (Beijing, China) Chloroauric acid (HAuCl_4_·4H_2_O), silver nitrate (AgNO_3_), sodium borohydride (NaBH_4_), nitric acid (HNO_3_), and hydrochloric acid (HCl) were purchased from Sinopharm Chemical Reagent Co., Ltd. (Shanghai, China). Cetyltrimethylammonium bromide (CTAB) and cetyltrimethylammonium chloride (CTAC) were bought from Macklin Biochemical Technology Co., Ltd. (Shanghai, China). Both 8-hydroxyquinoline and citrate were obtained from Aladdin Chemistry Reagent Co., Ltd. (Shanghai, China). Deoxynivalenol (DON), aflatoxin B_1_ (AFB_1_), aflatoxin M_1_ (AFM_1_), zearalenone (ZEN), and ochratoxin A (OTA) were supplied by the Academy of National Food and Strategic Reserves Administration (Beijing, China). Unless otherwise stated, all chemicals were purchased from commercial suppliers and used without further purification.

### 4.2. Synthesis of Au NBPs

A seed-mediated growth strategy was used to synthesize Au NBPs [29]. Primitively, CTAC (4 mL, 95 mM), HAuCl_4_ (4 mL, 0.5 mM), and HNO_3_ (72 μL, 250 mM) were mixed, followed by adding freshly prepared ice-cold NaBH_4_ (100 μL, 50 mM) under vigorous stirring for 1 min. The mixture color changed from light yellow to red–brown, then citrate (16 μL, 1 M) was added. After 90 min of the thermal treatment at 81 °C in an oil bath, the seed solution became reddish and then was stored at room temperature. For the synthesis of the growth solution, 125 μL of the prepared seed solution was added into the mixture of CTAB (40 mL, 47 mM), HAuCl_4_ (1 mL, 10 mM), AgNO_3_ (180 μL, 10 mM), and 8-hydroxyquinoline (400 μL, 0.4 M in ethanol) under vigorous stirring and left undisturbed at 45 °C for 15 min. After another 250 μL of 8-hydroxyquinoline was added and maintained at 45 °C for 15 min and 30 °C for 4 h, the Au NBPs were obtained. The as-synthesized Au NBPs were washed and redispersed in 0.1% CTAC (containing 1 mM HNO_3_). The washing process was repeated twice, and the Au NBPs were finally dispersed in 0.05% CTAB and stored at room temperature before use. Au NBPs with different aspect ratios were synthesized with various volumes of Au seeds. The morphology and size of the Au NBPs were characterized with transmission electron microscopy (TEM, JEOL JEM 2100F, Tokyo, Japan).

### 4.3. Preparation of IMBs

IMBs modified by DON mAb were prepared through covalent linkage [30,31]. Briefly, 100 mL of NHS-MBs were rinsed with absolute ethanol by quickly oscillating for 30 s, followed by the addition of 37.4 mL of DON mAb (10.71 mg/mL, purity > 96%) and 50 mL of reaction buffer (MES, pH = 6.0). The mixture was incubated under gentle stirring conditions at room temperature for 3 h, then the MBs were separated and suspended in 100 mL of blocking buffer (2% glycine in MES, pH = 7.2) for further incubation of 3 h. Finally, the precipitate was rinsed with washing buffer (0.1% Tween-20 in PBS) and suspended in 200 mL of PBS. The maximum capture capacity of the IMBs was analyzed by LC–MS/MS (1290-6470, Agilent, Santa Clara, CA, USA), the result showed that 100 μL of IMBs could capture 473.7 ng of DON.

### 4.4. Principle of HRP Catalyzed Etching of Au NBPs

HRP with gradient concentrations was used to catalyze the chromogenic reaction between TMB and H_2_O_2_. Then, the catalytic reaction was terminated by adding 2M HCl. The absorbance of the products was measured and the linear range of the catalytic reaction was investigated. Afterward, Au NBPs colloidal solution (Abs ≈ 1.5) with equal volume was introduced to the products. After 10 min, the UV–visible absorption spectra of Au NBPs in each well were measured by a microplate spectrophotometer (PerkinElmer, VICTOR Nivo, Waltham, MA, USA). The photographs of the reaction wells were taken with a smartphone (Mate30 pro, Huawei, Shenzhen, China). The reaction time of catalysis and etching and the concentration of CTAB were optimized.

### 4.5. Visual Detection of DON

The procedure of multicolor visual strategies for DON detection is illustrated in Figure 1. The process was carried out through an automated multiplex reaction system reported in our early work [32,33]. The reagents were encapsulated into the 7 wells of the auxiliary kit in sequence, including dilute solution (PBS, 4.8 mL), IMBs (600 μL), washing buffer (0.1% Tween-20 in PBS, 1 mL), HRP-linked DON antigen (600 μL), washing buffer (0.1% Tween-20 in PBS, 1 mL), TMB two-component substrate (100 μL), and Au NBPs (100 μL). The relevant parameters of the reaction program were edited as shown in Table S1. For DON detection, 200 μL of the sample was added into a dilute solution and the IMBs were transferred by a magnetic stick to capture the target. After immunoreaction, washing, enzyme catalysis, and Au NBP etching, the product was measured by a microplate spectrophotometer.

### 4.6. Selectivity of the Assay

A series of mycotoxins with similar chemical structures were chosen to investigate the selectivity of the proposed detection method. Standard solutions of DON, AFB_1_, AFM_1_, ZEN, and OTA were introduced into the sample well, respectively, and the concentrations of all mycotoxins were 1000 ng/mL. The plasma absorption peak and color of Au NBPs were recorded.

### 4.7. Validation of the Method

The proposed analytical method was evaluated to detect naturally contaminated samples of wheat and maize. The grated sample (5.0 ± 0.005 g) was extracted with 20.0 mL of PEG 8000 solution (5%) by vigorous oscillation for 5 min (wheat) and 3 min (maize), respectively. After centrifuging at 7000 rpm for 5 min, 200 μL of supernatant was added to the auxiliary kit and reacted according to the edited program. The average, recovery, and relative standard deviation (RSD) of the results were evaluated.

## Figures and Tables

**Table 1:** Recoveries and corresponding RSD of the developed method for detecting DON in naturally contaminated wheat and maize (*n* = 4).

Sample	State Value/(μg/kg)	Detected Average/(μg/kg)	Recovery/%	RSD/%
Wheat	200 ± 30	211.4	105.7	11.80
	400 ± 60	418.0	104.5	11.61
	610 ± 92	622.1	101.9	9.59
	820 ± 98.4	820.7	100.1	8.67
	1023 ± 153	1002	97.98	4.10
	1033 ± 199	1263	94.74	9.15
	1606 ± 241	1604	99.85	9.61
	1845 ± 227	1759	95.33	4.06
Maize	231 ± 34	225.4	97.57	5.15
	462 ± 43	443.9	96.09	10.03
	770 ± 115	816.8	106.1	8.70
	1077 ± 161	1157	107.5	7.12
	1577 ± 236	1477	93.65	6.56
	2076 ± 294	2073	99.85	9.94

## Data Availability

Data available on request from the authors.

## Supplementary Materials

The following supporting information can be downloaded at: https://www.mdpi.com/article/10.3390/toxins15060351/s1. Table S1: The step sequence and time of the automatic clean-up procedure; Table S2: A comparison of the performance of different analytical methods for the detection of DON; Figure S1: (A) UV–vis spectrum of Au NBPs synthesized with various amount of Au seeds. (B) Relationship between volume of Au seeds and LSPR peak of Au NBPs; Figure S2: (A) UV–vis spectrum of TMB^2+^ catalyzed with various concentrations of HRP. (B)The relationship between the absorbance of TMB^2+^ at 452 nm and the concentration of HRP before the addition of Au NBPs; Figure S3: Effects of the experimental conditions on Δλ of Au NBPs: catalytic reaction time (A), etching reaction time (B), and the concentration of CTAB (C, D); Figure S4: Specificity of the multicolor visual detection method for DON.
