# Supplementary material for: Multicolor Visual Detection of Deoxynivalenol in Grain Based on Magnetic Immunoassay and Enzymatic Etching of Plasmonic Gold Nanobipyramids
Source: Toxins (Basel). 2023 May 23;15(6):351. doi: 10.3390/toxins15060351 (PMC10304167; doi:10.3390/toxins15060351)
Supplement: Supplementary file 1 [file toxins-15-00351-s001.zip › toxins-2379563-supplementary.pdf]

Supplementary Materials: Multicolor visual detection of deoxynivalenol in grain based on magnetic immunoassay and enzymatic etching of plasmonic gold nanobipyramids

Rui Guo, Yue Ji, Jinnan Chen, Jin Ye, Baoxia Ni, Li Li, Yongtan Yang

Table S1. The step sequence and time of the automatic clean-up procedure.

No.	Step	Well	Reagent	Mixing Time/min	Collection Time/min	Mixing Frequency/Hz
1	Adsorption	2	IMBs	1	2	8
2	Capture	1	PBS+DON	5	5	8
3	Wash 1	3	0.1% PBST	1	2	8
4	Binding	4	HRP-DON	5	2	8
5	Wash 2	5	0.1% PBST	1	2	8
6	Catalysis	6	TMB/H_2O_2	5	2	8
7	Collection	2	PBS	1	0	8
8	Etching	7	Au NBPs	5	0	8

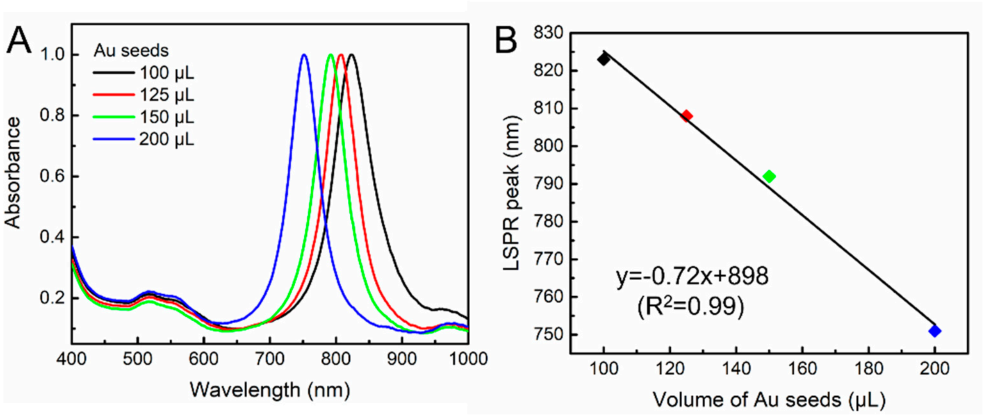

Figure S1. (**A**) UV-vis spectrum of Au NBPs synthesized with various amount of Au seeds. （**B**）Relationship between volume of Au seeds and LSPR peak of Au NBPs.

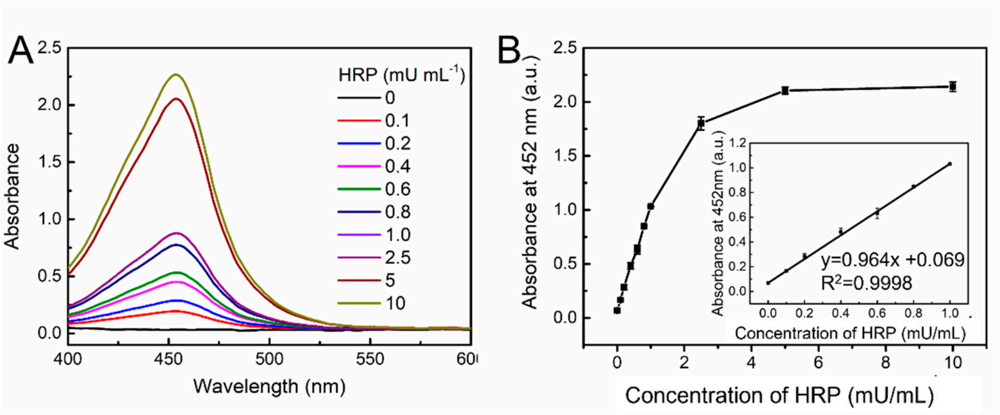

Figure S2. （**A**）UV-vis spectrum of TMB^{2+} catalyzed with various concentration of HRP. （**B**）The relationship between the absorbance of TMB^{2+} at 452 nm and the concentration of HRP before the addition of Au NBPs.

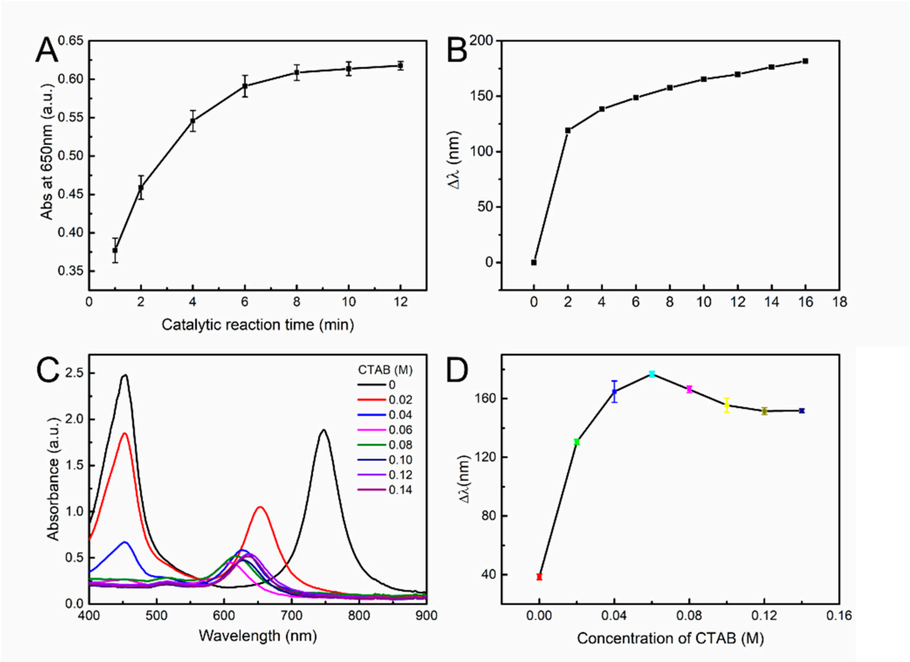

Figure S3. Effects of the experimental conditions on $\Delta\lambda$ of Au NBPs: Catalytic reaction time (**A**), etching reaction time(**B**), and the concentration of CTAB (**C**, **D**).

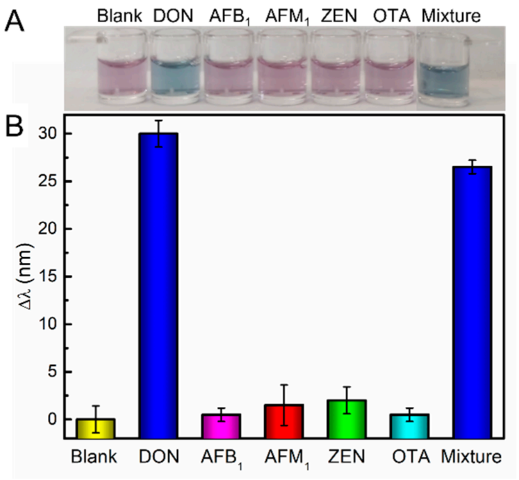

Figure S4. Specificity of the multicolor visual detection method for DON.

Table S2. A comparison of the performance of different analytical methods for detection of DON.

Method	LOD/(ng/mL)	Linear range /(ng/mL)	Recovery rate/%	RSD /%	Operation	Time /min	Visualization	Reference
Competitive direct enzyme-linked immunosorbent assay	30	150 - 480	70 - 100	<11.45	tedious	>60	no	10
Colloidal gold immunochromatographic strip	50	0 - 50	80 - 90	/	convenient	10	yes	11
Quantum dot nanobead-based fluorescent immunochromatographic assay	2.97	0.465 - 16.19	82.65 – 116.35	<12.03	convenient	25	yes	12
Multicolor visual method	57.93	0 - 2000	93.65 - 107.5	<11.8	automation	40	yes	this work
